# Non-Coding RNAs in Oral Diseases: From Pathogenesis to Clinical Translation

**DOI:** 10.3390/ijms27177832

**Published:** 2026-09-01

**Authors:** Letong Huang, Hailong Zhang, Yi Huang, Ting Lu, Maoyuan Zeng, Haobin Wu, Junhao An, Bin Chen, Jianguo Liu, Qunli Ren

**Affiliations:** 1 Key Laboratory of Oral Disease Research of Guizhou Education Department, School of Stomatology, Zunyi Medical University, Zunyi 563000, China; zmuhlt0530@163.com (L.H.); 15208650346@163.com (Y.H.); ashley16888@163.com (T.L.); 15519640000@163.com (M.Z.); 18585334620@163.com (J.A.); chenbin@zmu.edu.cn (B.C.); 2 Key Laboratory of Cancer Prevention and Treatment of Guizhou Province, Zunyi Medical University, Zunyi 563000, China; hailongzyolo@163.com; 3 Key Laboratory of Microbial Resources and Drug Development of Guizhou Education Department, School of Stomatology, Zunyi Medical University, Zunyi 563000, China; 18212310142@163.com

**Keywords:** non-coding RNAs, oral diseases, periodontitis, oral squamous cell carcinoma, competing endogenous RNA networks, liquid biopsy, micropeptide-encoding ncRNAs, translational medicine

## Abstract

Oral diseases represent a major global health burden, and non-coding RNAs (ncRNAs) have emerged as pivotal regulators in their pathogenesis, offering new avenues for diagnosis and therapy. This review synthesizes current knowledge on ncRNAs—including miRNAs, lncRNAs, and circRNAs—across a spectrum of oral conditions, encompassing periodontitis, oral squamous cell carcinoma, oral submucous fibrosis, oral lichen planus, oral leukoplakia, and chronic orofacial pain. Mechanistically, ncRNAs function through competing endogenous RNA networks, immune–inflammatory cascades, metabolic reprogramming, and fibroblast activation; notably, recent discoveries have revealed that certain circRNAs and lncRNAs encode functional micropeptides, adding an additional layer of regulatory complexity in oral pathology. On the translational front, salivary and circulating ncRNAs have shown promise as non-invasive biomarkers. However, major bottlenecks remain, including insufficient longitudinal validation, marked technical heterogeneity across studies, and delivery challenges specific to the oral microenvironment. By critically evaluating mechanistic insights alongside these translational barriers, this review identifies critical knowledge gaps and proposes prioritized strategies to facilitate the clinical integration of ncRNA-based precision dentistry.

## 1. Introduction

Oral diseases encompass a heterogeneous group of pathological conditions that arise in multiple anatomical and cellular microenvironments, including dental hard tissues, dental pulp, the periodontium, the oral mucosa, the jawbone, and peri-implant tissues. Periodontitis is primarily characterized by plaque biofilm-induced chronic inflammation and destruction of the periodontal supporting tissues; pulpitis and periapical periodontitis involve infection, immune responses, neurovascular regulation, and hard tissue repair; peri-implantitis manifests as inflammatory destruction of the soft and hard tissues around dental implants; and oral potentially malignant disorders and oral squamous cell carcinoma involve inflammation, genetic damage, epigenetic remodeling, and alterations in the tumor microenvironment [1,2,3,4,5,6]. Although these diseases present distinct clinical features, they share several important pathological bases, including microbial dysbiosis, epithelial barrier disruption, aberrant immune cell recruitment, sustained release of inflammatory cytokines, epigenetic remodeling, altered cell death pathways, and impaired tissue regeneration.

**Figure 1:**
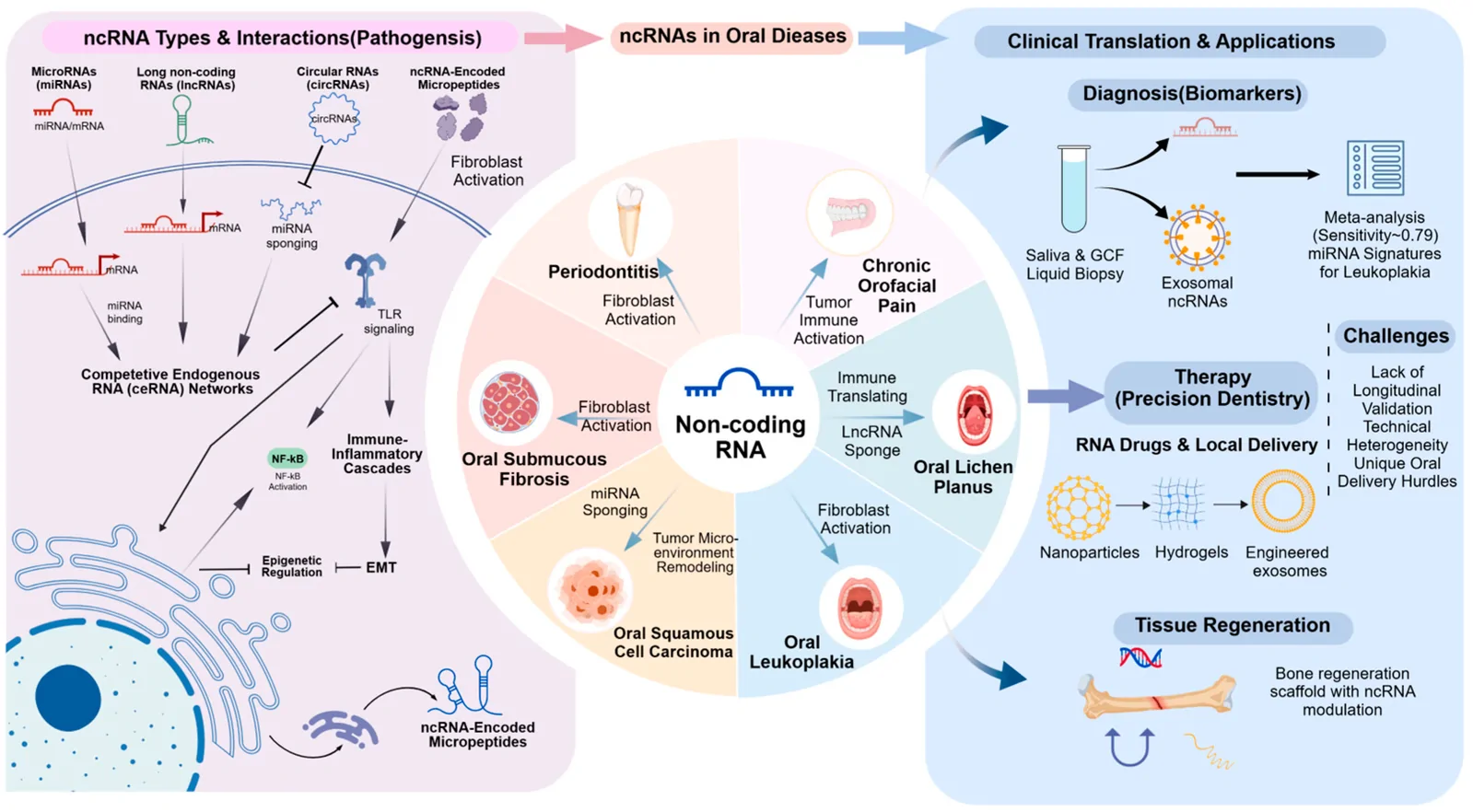
Multifaceted roles of non-coding RNAs in the pathogenesis and clinical translation of oral diseases. Created with BioGDP.com. From a clinical translation perspective, oral diseases offer a natural advantage for liquid biopsy. Saliva, gingival crevicular fluid (GCF), peri-implant sulcular fluid (PISF), pulp tissue, and exfoliated oral mucosal cells are all relatively accessible sample sources. Among these, salivary and exosomal ncRNAs have garnered attention due to their non-invasive nature, suitability for repeated collection, and dynamic monitoring capabilities [11,12,13,14]. However, existing studies often suffer from limitations such as focus on a single disease type, insufficient validation of biomarkers, and a disconnect between mechanistic research and clinical application. Therefore, it is necessary to systematically review the evidence and common mechanisms of ncRNA applications across the spectrum of oral diseases.

Traditional research on oral diseases has largely focused on protein-coding genes, inflammatory cytokines, microbial composition, and histopathological changes. With the development of high-throughput sequencing, single-cell transcriptomics, spatial transcriptomics, and extracellular vesicle analysis technologies, a large number of RNA molecules that do not encode conventional proteins have been shown to participate in oral tissue homeostasis and disease progression [7,8,9,10]. Non-coding RNAs (ncRNAs) can play fine-tuning regulatory roles in gene expression regulatory networks, and their expression is cell type-, disease stage-, and microenvironment-dependent. Compared with single inflammatory cytokines or protein markers, ncRNAs are better suited to explain the continuous regulatory relationships among “local microecology–immune inflammation–tissue destruction–repair failure” in oral diseases (Figure 1).

## 2. Types of ncRNAs and Oral Diseases

### 2.1. Major Types of ncRNAs

miRNAs are a class of small RNAs, approximately 19–25 nucleotides in length, that primarily inhibit translation or promote mRNA degradation by complementary binding to target mRNAs. Numerous studies have shown that miRNAs can regulate the release of inflammatory cytokines, the osteoblast–osteoclast balance, and the migration and apoptosis of periodontal ligament cells in periodontitis, as well as influence the proliferation, invasion, epithelial–mesenchymal transition (EMT), and treatment sensitivity of oral cancer cells [15,16,17,18]. At the clinical application level, due to their short length, mature detection technologies, and relative stability in body fluids, miRNAs represent a well-evidenced class of ncRNAs in the diagnosis and prognosis of oral diseases. It should also be noted that both -3p and -5p mature forms exist, and they typically regulate distinct target mRNAs due to different seed sequences, although partial overlap may occur.

lncRNAs are typically defined as RNA molecules longer than 200 nucleotides that lack classical protein-coding capacity. Their regulatory mechanisms are complex, involving chromatin modification, transcriptional regulation, modulation of mRNA stability, miRNA sponging, and interactions with RNA-binding proteins, thereby enabling their participation in disease processes. Functionally, lncRNAs can act as competing endogenous RNAs (ceRNAs) to sponge miRNAs, as scaffolds for protein complexes, or as guides to recruit chromatin-modifying enzymes to specific genomic loci, thereby influencing gene expression at multiple levels. In oral diseases, lncRNAs have been reported to be involved in inflammatory injury of periodontal ligament cells, impaired cementoblast mineralization, immune responses in pulpitis, and metastasis and drug resistance in oral cancer [19,20,21,22,23,24,25]. Notably, the tissue- and cell-specific expression patterns of lncRNAs make them attractive candidates for disease subtyping and precision therapeutic targeting. Due to their strong tissue and cell specificity, lncRNAs hold theoretical advantages as therapeutic targets, but this same specificity also increases the difficulty of mechanistic interpretation and clinical standardization.

circRNAs are generated by back-splicing of pre-mRNA, forming covalently closed loop structures. Lacking a 5′ cap and 3′ poly(A) tail, they are relatively resistant to exonuclease digestion. Their circular conformation confers high stability in body fluids, making them promising non-invasive biomarker candidates. circRNAs can act as miRNA sponges, interact with RNA-binding proteins, and regulate transcription and splicing, and some circRNAs have the potential to encode small peptides [26,27,28,29]. Furthermore, circRNAs have been shown to regulate gene expression not only through miRNA sequestration but also via protein scaffolding, modulation of parental gene transcription, and, in some cases, translation into functional micropeptides. In periodontitis, peri-implantitis, and oral cancer, circRNA-associated competing endogenous RNA (ceRNA) networks have gradually become an important approach to explaining disease heterogeneity and constructing prognostic models [29,30,31,32,33].

It is important to note that circRNA research is highly sensitive to detection workflows and bioinformatics analysis. The identification of circRNAs relies on detecting back-splice junctions and is susceptible to influences such as sequencing depth, RNase R treatment, read length, alignment algorithms, false-positive back-splicing events, and host gene expression levels [34,35,36]. Chen et al. [34] pointed out, from a biogenesis perspective, that circRNA production is co-regulated by cis-elements and trans-acting splicing factors; Szabo and Salzman [35] emphasized that circRNA detection requires stringent differentiation of true back-splicing events from linear transcripts or sequencing artifacts; and Chen et al. [36] further summarized bioinformatics tools for circRNA discovery, annotation, quantification, functional prediction, and database integration. For oral disease research, in the absence of a unified circRNA identification pipeline and independent experimental validation, mechanistic inferences based on ceRNA networks are prone to over-interpretation.

Exosomal ncRNAs are not a distinct RNA type but rather a collection of RNA molecules encapsulated and transported by extracellular vesicles. Exosomes can transfer miRNAs, lncRNAs, and circRNAs among oral epithelial cells, fibroblasts, immune cells, endothelial cells, periodontal ligament cells, and tumor cells, thereby participating in inflammatory amplification, tissue repair, angiogenesis, immune evasion, and pre-metastatic niche formation [37,38]. Because the exosomal membrane protects internal RNAs from degradation, exosomal ncRNAs are considered important vehicles for liquid biopsy in oral diseases.

### 2.2. Types of Oral Diseases and ncRNA

**Periodontitis** is currently one of the most active fields of ncRNA research in oral diseases. Reviews by Adamouli et al. [1] and Feng et al. [2] indicate that miRNAs, lncRNAs, and circRNAs collectively participate in immune inflammation, bone homeostasis imbalance, and cell proliferation and apoptosis in periodontitis. In specific studies, miR-671-5p has been reported to be involved in the diagnosis and therapeutic target exploration of periodontitis via THBS1 [38]; EPB41L4A-AS1 regulates periodontal ligament cell injury and osteogenic differentiation through the miR-214-3p/YAP1 axis [20]; AC114812 modulates the inflammatory response of periodontal ligament cells via the miR-181a-5p/SPP1 axis [21]; and circ_0138959 is associated with pyroptosis of gingival fibroblasts [30].

**Peri-implantitis** shares a similar microbial inflammatory background with periodontitis, but its tissue structure, material interface, and immune response characteristics are not identical. Zhou et al. [31] compared mRNA, miRNA, and circRNA expression networks in periodontitis and peri-implantitis, suggesting that circRNA-mediated ceRNA regulation exhibits disease context-dependent differences. Such studies indicate that ncRNAs can serve as biomarkers not only for a single disease but also for comparing the molecular heterogeneity of different oral inflammatory diseases.

In **pulpitis** and periapical diseases, infectious stimuli induce complex reactions involving dental pulp cells, immune cells, and neurovascular components. Recent reviews of lncRNAs and circRNAs in pulpitis have pointed out that these molecules may be involved in the release of inflammatory mediators, apoptosis, pyroptosis, angiogenesis, and pulp repair [22]. Furthermore, in a *P. gingivalis*-induced periapical periodontitis model, the Platr3/NUDT21/NF-κB axis was shown to be associated with impaired cementoblast mineralization [19], suggesting that ncRNAs act as a link between odontogenic inflammation and hard tissue regeneration disorders.

Oral potentially malignant disorders and oral cancer represent other major directions for ncRNA application research. In oral squamous cell carcinoma (OSCC), miR-21, miR-31, miR-375, HOTAIR, MALAT1, H19, PVT1, and various circRNAs are associated with tumor proliferation, invasion, metastasis, prognosis, and treatment resistance [16,17,18,23,24,25,26,27,28].

Beyond the diseases mentioned above, ncRNAs are also used to explain the osteogenic differentiation of periodontal ligament-derived cells, bone homeostasis abnormalities, and oral tissue engineering repair. Sufianov et al. [39] summarized the role of ncRNAs in the osteogenic differentiation of human periodontal ligament-derived cells; although the review of osteoporosis by Ko et al. [40] is not focused on oral diseases, its mechanisms of miRNA/lncRNA regulation of the osteoblast–osteoclast balance can serve as a reference for studies on alveolar bone resorption and regeneration.

## 3. Key Mechanisms of ncRNA Action

### 3.1. Microecological Imbalance, Immune Inflammation, and ncRNA Regulation

This mechanism is particularly prominent in periodontitis, peri-implantitis, and pulpitis, where microbial dysbiosis drives chronic inflammatory responses. The oral cavity is one of the sites with the highest microbial density in the human body. Dental plaque biofilm, saliva, gingival crevicular fluid, and mucosal immunity collectively shape the local microenvironment. In periodontitis and peri-implantitis, microbial dysbiosis can induce the activation of Toll-like receptors (TLRs), NF-κB, the NLRP3 inflammasome, IL-1β, IL-6, TNF-α, and the RANKL/OPG axis, ultimately leading to inflammatory amplification and alveolar bone resorption [1,2,41,42]. ncRNAs can regulate this process by targeting key molecules in inflammatory pathways. For example, the Platr3/NUDT21/NF-κB axis is involved in *P. gingivalis*-induced odontogenic inflammation and impaired mineralization [19]; circ_0138959 participates in the pyroptosis of gingival fibroblasts via the miR-527/caspase-5 axis [30].

Single-cell studies further reveal that the oral inflammatory microenvironment is not driven by a single cell population. Qiu et al. [43] constructed a single-cell atlas of the human gingiva and identified cell subpopulations associated with neutrophil extracellular traps (NETs) that participate in periodontal immunopathology. NETs are linked to chronic inflammation, tissue damage, and tumor microenvironment remodeling. Although direct evidence is still limited, it is plausible that inflammation-related ncRNAs may influence oral disease progression by modulating communication among neutrophils, macrophages, fibroblasts, and epithelial cells.

Metatranscriptomic studies also suggest that the microbiota in periodontitis exhibit not only compositional changes but also alterations in metabolic functions and potential ncRNA expression. Ram-Mohan and Meyer [44] identified shared metabolic functional shifts in periodontitis-associated microbiota and recognized potential disease-related ncRNAs. Such studies extend ncRNA analysis from host cells to the microecological level, providing new entry points for understanding microbiota–host interactions.

The fine-tuning of innate immune signaling by miRNAs is a crucial mechanism connecting infection and inflammatory injury. O’Neill et al. [45] proposed that miRNAs act as “fine-tuners” of TLR signaling pathways, affecting inflammatory intensity by modulating inflammatory cytokines, negative feedback inhibitors, and immune cell activation thresholds. Luan et al. [46] further summarized, from the perspective of periodontal health and disease, the regulation of neutrophil, macrophage, dendritic cell, T-cell, and B-cell functions by miRNAs, noting that miRNAs such as miR-15a, miR-29b, miR-125a, miR-146a, miR-148a, and miR-223 have repeatedly been found to be abnormally expressed in periodontitis [47]. This evidence suggests that miRNA changes in periodontitis are not merely incidental to inflammation but may determine the intensity of the host’s immune response to plaque biofilm and the degree of tissue destruction.

Genetic background is also an important dimension for explaining individual differences in oral diseases. A review of the genetics of periodontitis by Schaefer [48] pointed out that host genetic factors can influence immune receptors, signal transduction, thresholds of inflammatory responses, and susceptibility to oral microorganisms. Variations in ncRNA loci, miRNA binding sites, and epigenetic regulation may collectively contribute to disease susceptibility. Recently, Polizzi et al. [49] analyzed, from a translational medicine perspective, the relationships between periodontitis-related miRNAs and disease severity, systemic inflammation, and potential systemic impacts, suggesting that local oral ncRNA abnormalities may not only reflect local pathology but may also participate in oral–systemic disease axes.

### 3.2. ceRNA Networks and Cell Fate Regulation

The ceRNA network is a conserved regulatory mechanism across multiple oral diseases, with particularly well-documented roles in periodontitis and oral squamous cell carcinoma. The ceRNA network is currently one of the most common mechanistic frameworks in ncRNA research on oral diseases. lncRNAs and circRNAs can competitively bind to miRNAs, relieving the inhibition of target mRNAs by miRNAs, thereby affecting inflammation, apoptosis, pyroptosis, osteogenic differentiation, EMT, and tumor metastasis [50]. In periodontitis, the EPB41L4A-AS1/miR-214-3p/YAP1 axis and the AC114812/miR-181a-5p/SPP1 axis have been reported to be involved in periodontal ligament cell injury, osteogenic differentiation, and inflammatory responses, respectively [20,21]. In peri-implantitis, circRNA-mediated ceRNA networks exhibit disease specificity, potentially explaining the molecular differences from periodontitis [31].

In oral cancer, ceRNA networks are closely related to tumor heterogeneity and treatment resistance. lncRNAs such as HOTAIR, MALAT1, H19, and PVT1 can regulate EMT, cancer stem cell properties, metabolic reprogramming, and drug tolerance through miRNA sponging or epigenetic mechanisms [23,24,25,51,52,53,54]. circRNAs can modulate pathways such as VEGFA, MMPs, PI3K/AKT, and Wnt/β-catenin via miRNA sponging and are associated with OSCC prognosis and immune infiltration [26,27,28,29,33,55]. These studies demonstrate that ncRNAs are not simple upstream or downstream molecules but nodes within multi-layered regulatory networks.

Alterations in cell death pathways also represent an important mechanism by which ncRNAs participate in oral diseases. In periodontitis, pyroptosis, apoptosis, and autophagy can affect the functions of gingival fibroblasts, periodontal ligament cells, and immune cells; in oral cancer, evasion of apoptosis and altered autophagy are often associated with treatment resistance. circRNAs and lncRNAs can influence cell fate by regulating the caspase family, BCL-2 family, NLRP3 inflammasome, and AKT/mTOR pathway [30,51,56].

### 3.3. Exosome-Mediated Intercellular Communication

Exosome-mediated communication is a critical mechanism in both inflammatory oral diseases (periodontitis and pulpitis) and oral cancer, though the specific cargo and functional consequences differ by disease context. Exosomes are crucial information carriers connecting local cell populations in the oral cavity. In periodontitis and pulpitis, inflammatory stimuli can alter the composition of miRNAs, lncRNAs, and circRNAs within extracellular vesicles, thereby influencing immune cell recruitment, fibroblast activation, and tissue repair. In oral cancer, tumor-derived exosomal ncRNAs promote cancer-associated fibroblast formation, angiogenesis, immune evasion, and pre-metastatic niche establishment [37,38]. Therefore, exosomal ncRNAs are not only part of the disease mechanism but also important vehicles for liquid biopsy and drug delivery.

Beyond exosomes, apoptotic bodies and apoptotic vesicles should also be incorporated into the framework of extracellular vesicle research in oral diseases. Li et al. [57] found that mesenchymal stem cell-derived apoptotic bodies, enriched in miR-223-3p, inhibit osteoclast differentiation and function, thereby alleviating alveolar bone destruction, suggesting that apoptotic bodies are not merely cellular debris but may play roles in tissue protection and regeneration regulation. Conversely, Xiao et al. [58] reported that macrophage-derived apoptotic vesicles exacerbate periodontitis-associated tissue destruction, indicating that extracellular vesicles from different sources and at different pathological stages may have opposing effects. Therefore, future studies on liquid biopsy and therapeutic delivery in oral diseases should not simply classify all vesicles as exosomes but should distinguish the sources and functions of exosomes, microvesicles, and apoptotic bodies and their RNA cargo.

Salivary exosomes are of particular interest in oral disease research. Saliva is in direct contact with the oral mucosa, periodontal tissues, and tumor lesions, allowing it to relatively directly reflect local microenvironmental changes. Compared with tissue biopsy, saliva collection is non-invasive and repeatable, making it suitable for disease screening and dynamic monitoring [11,12,13,14]. However, salivary exosomes are significantly influenced by oral hygiene, food intake, periodontitis, bleeding, and microbial contamination, necessitating the establishment of rigorous standardized protocols (Table 1 and Table 2).

## 4. Clinical Applications and Translational Challenges

### 4.1. Diagnosis, Prognosis, and Disease Monitoring

The diagnostic and prognostic utility of ncRNAs varies considerably across oral diseases. In oral cancer, ncRNA biomarkers have been extensively validated and show promise for clinical translation; in periodontitis, pulpitis, and peri-implantitis, however, the evidence remains largely exploratory and requires further validation. The clinical application of ncRNAs in oral diseases is first reflected in biomarker development. In terms of periodontitis, Adamouli et al. [1] systematically reviewed the expression patterns of ncRNAs and found that, while miRNAs—particularly miR-146a and miR-142-3p—show relatively consistent upregulation, the evidence for lncRNAs and circRNAs remains inconclusive due to methodological heterogeneity and small sample sizes. Feng et al. [2] further summarized the evidence, stating that ncRNAs regulate immune inflammation, bone homeostasis, and cell proliferation in periodontitis, but noted that most studies lack standardized detection methods and clinical validation. In terms of peri-implantitis, Zhou et al. [31] demonstrated that circRNA-mediated ceRNA regulation exhibits disease context-dependent differences from periodontitis, though this finding requires further validation. In terms of pulpitis, Fuchen-Ramos et al. [22] conducted a review and found that lncRNAs and circRNAs have been validated in in vitro models and patient samples, but they emphasized that nearly all findings were derived from cellular culture systems without animal model or large cohort validation and that no genome-wide circRNA expression profiling in human pulpitis has been reported.

In oral cancer, by contrast, ncRNA biomarker research is considerably more mature. In terms of miRNAs, Liu et al. [16] validated salivary miR-31 as a non-invasive biomarker that decreases after tumor resection; Lin et al. [17] confirmed miR-31 upregulation in plasma, saliva, and tumor tissue with associations with TNM staging, metastasis, and chemotherapy resistance, as well as demonstrating that miR-203 and miR-205 achieve 100% sensitivity and specificity in detecting HNSCC lymph node metastases; and Sun et al. [18] confirmed the prognostic value of multiple miRNAs via a meta-analysis. In terms of lncRNAs, Troiano et al. [23] conducted a meta-analysis showing that HOTAIR overexpression is associated with poor overall survival (HR = 1.90), advanced tumor stage (OR = 3.44), and lymph node metastasis (OR = 3.31); Zhou et al. [24] validated MALAT1 as an independent prognostic factor promoting EMT through the β-catenin and NF-κB pathways; and Wang et al. [25] showed that PVT1 upregulation correlates with T classification, clinical stage, and lymph node metastasis via Wnt/β-catenin activation. In terms of circRNAs, Fan et al. [27] conducted a comprehensive review and found that multiple circRNAs serve as diagnostic and prognostic biomarkers in OSCC, with salivary circ_0001874 and circ_0001971 achieving an AUC of 0.922 when combined. It should be noted, however, that these salivary circRNA findings were derived from a single-center discovery cohort without independent external validation, and their back-splice junctions have not been systematically confirmed by RNase R resistance assays or Sanger sequencing—criteria that are emphasized in Section 2.1 for rigorous circRNA identification [34,35,36]. Thus, while promising, these results should be considered exploratory rather than clinically validated.

However, it is critical to recognize that periodontitis, pulpitis, and peri-implantitis are far more prevalent in the general population than oral cancer, yet their ncRNA biomarker development remains largely at the discovery stage with limited clinical validation. The diagnostic logic also differs fundamentally: non-malignant conditions primarily reflect inflammation and tissue homeostasis, whereas oral cancer involves malignant transformation. Although a definitive panel cannot yet be recommended for routine testing, the most consistently reported candidates—including miR-146a for periodontitis and miR-31, miR-375, MALAT1, HOTAIR, and salivary circ_0001874/circ_0001971 for OSCC—represent priority targets for future multicenter validation. Therefore, future research should prioritize standardized detection protocols, multicenter prospective cohorts, and independent large-scale validation to translate ncRNA biomarkers into clinical practice for these more common but understudied oral diseases.

### 4.2. Therapeutic Targets and Regenerative Medicine Applications

The therapeutic potential of ncRNAs has been investigated across the spectrum of oral diseases, with strategies tailored to the specific pathology of each condition: immunomodulation in periodontitis, pulp regeneration in pulpitis, and reversing drug resistance in oral cancer. ncRNAs can serve as therapeutic targets, with theoretical strategies including miRNA mimics, anti-miRNA oligonucleotides, siRNAs, antisense oligonucleotides, small-molecule modulators, CRISPR-based regulation, nanocarriers, and engineered exosome delivery. In periodontitis, modulating inflammation-related miRNAs or lncRNAs may alleviate immune damage and promote periodontal tissue repair; in pulpitis, intervening in ncRNA networks may improve inflammation control and pulp regeneration; and in oral cancer, inhibiting oncogenic miRNAs, lncRNAs, or circRNAs may reverse EMT and treatment resistance [51,56,59].

Oral diseases offer an advantage for local drug delivery. The periodontal pocket, root canal system, oral mucosa, and tumor surgical site can all serve as local delivery sites, providing application space for RNA drugs, nanoparticles, hydrogels, and degradable scaffolds. In periodontal tissue regeneration research, the regulation of the osteogenic differentiation, fibroblastic differentiation, and mineralization capacity of periodontal ligament-derived cells by ncRNAs is particularly important [40,41]. Combining ncRNA regulation with biomaterials, stem cells, and local sustained-release systems could propel oral regenerative medicine from empirical repair toward molecularly precise regulation.

However, therapeutic applications still face significant obstacles. RNA drugs are prone to degradation in vivo, with insufficient delivery efficiency and cell specificity; antisense oligonucleotides or siRNAs may cause off-target effects and immune responses; and the local oral environment, including salivary flow, enzymatic degradation, plaque barriers, and mechanical stimulation, may all affect drug stability. For cancer therapy, tumor heterogeneity, the immunosuppressive microenvironment, and combination treatment regimens must also be considered.

Oral diseases should also be understood within the context of oral–systemic interactions. Xie et al. [60] found that *P. gingivalis* can induce apoptosis of vascular smooth muscle cells via the TLR2 pathway and promote the release of miR-143/145, which is then taken up by macrophages, thereby inhibiting macrophage clearance of apoptotic cells and exacerbating atherosclerosis. This study suggests that oral pathogen-induced ncRNA and extracellular vesicle signals may transcend local tissue boundaries and participate in systemic inflammation and cardiovascular risk. For high-impact journals, such evidence helps extend the application of ncRNAs in oral diseases from local diagnosis and treatment to a translational framework of oral–systemic medicine.

### 4.3. Translational Difficulties and Evidence Boundaries

The translational barriers discussed below apply broadly across periodontitis, pulpitis, peri-implantitis, and oral cancer, though their relative importance differs by disease. Current ncRNA research suffers from several common problems. First, disease definitions and sample sources are not uniform. Periodontitis, peri-implantitis, pulpitis, and oral cancer each have different stages, classifications, and disease courses; without strict clinical stratification, differential expression results may be difficult to interpret. Second, detection platforms vary considerably. Differences in sensitivity, normalization methods, and data processing workflows among qRT-PCR, microarrays, RNA-seq, small RNA-seq, and exosome sequencing lead to poor comparability across studies.

Methodological reproducibility is key to whether a study can be discussed in the context of top-tier journals. For circRNAs, differential expression results need to be validated by RNase R resistance, divergent/convergent primer design, and Sanger sequencing to confirm back-splice junctions, as well as preferably combined with absolute quantification or independent cohort validation [34,35,36]. For extracellular vesicle ncRNAs, studies should report vesicle isolation methods, size, morphology, marker proteins, RNA quality control, and contamination assessment. If conclusions are based solely on publicly predicted ceRNA networks without sample validation and functional rescue experiments, they are more suitable as hypothesis-generating evidence rather than as mechanistic evidence.

Third, the depth of mechanistic validation is often insufficient. Some studies only report differential ncRNA expression and target gene predictions, lacking dual-luciferase validation, RNA pull-down, RIP, rescue experiments, animal models, and clinical cohort validation. Fourth, clinical translation evaluation is inadequate. Biomarker studies need to report sensitivity, specificity, AUC, positive predictive value, negative predictive value, independent validation cohorts, and cost-effectiveness, whereas therapeutic target studies must address delivery, safety, and long-term efficacy.

## 5. Non-Coding RNA-Encoded Micropeptides in Oral Diseases: Emerging Research Directions and Therapeutic Potential

This section is presented as a forward-looking perspective rather than a summary of established evidence. Unlike the circRNA validation criteria discussed in Section 2.1—which require RNase R resistance, divergent primer validation, and Sanger sequencing of back-splice junctions—the micropeptide-encoding potential discussed here has not yet been experimentally confirmed in oral diseases. Readers should therefore interpret the following as hypothesis-generating observations that await rigorous experimental validation.

In recent years, the field of non-coding RNA (ncRNA) research has undergone a paradigm shift: long non-coding RNAs (lncRNAs) and circular RNAs (circRNAs), traditionally considered “non-coding,” have been demonstrated to harbor micropeptide-encoding capacity, profoundly reshaping our understanding of genomic function [61]. However, direct experimental evidence for such coding events in oral diseases remains scarce. This section is therefore presented as a forward-looking perspective, highlighting hypothetical opportunities and open questions rather than established conclusions. In the context of periodontitis, high-throughput sequencing and single-cell technologies have deepened ncRNA functional studies. Recent reviews have explicitly identified ncRNA-encoded peptides or proteins as an emerging direction for mechanistic dissection and clinical translation in periodontitis, systematically summarizing the functions, mechanisms, and diagnostic/therapeutic potential of ncRNAs in this disease [2]. Meanwhile, expression profiling of circRNAs in gingival tissues from periodontitis patients—including associations with Redondoviridae infection—has provided a data foundation for subsequent validation of their coding potential [62]. In pulpitis research, microarray and single-cell RNA sequencing have been employed to evaluate DNA damage responses in inflamed dental pulp, and the construction of circRNA/lncRNA–miRNA–mRNA regulatory networks has laid the groundwork for mining potentially coding-competent ncRNAs [63].

Oral squamous cell carcinoma (OSCC), a highly prevalent malignancy in the head and neck region, has been a particularly active area of ncRNA investigation. CircRNF13 is upregulated in oral cancer tissues and cells, correlates positively with tumor grade and stage, and promotes tumor progression in both in vitro and in vivo models [64]; circFNDC3B is significantly elevated in OSCC and closely associated with lymph node metastasis [65]. Although the current literature has not yet explicitly reported whether these circRNAs directly encode functional micropeptides, a consensus in the field holds that the discovery of circRNA-encoded peptides is attracting widespread attention and that these peptides play critical roles in cancer development and immune responses [66]. To inform future oral disease research, we briefly note that lncRNA-encoded micropeptides have been reported to exert either tumor-suppressive or oncogenic functions in certain other cancers (e.g., AC115619-22aa in hepatocellular carcinoma [67]); however, such observations should be viewed as hypotheses for oral cancers rather than direct evidence [68]. We also acknowledge that functional mechanisms characterized in other systems—such as signaling modulation, metabolic reprogramming, or epigenetic interference—offer valuable mechanistic hypotheses, but they require direct validation in oral pathological settings. Advances in computational tools and experimental methods are accelerating the prediction and validation of the coding potential of oral cancer-related ncRNAs [69].

The functional mechanisms of micropeptides are diverse: they can modulate signaling pathways (e.g., MIAC inhibits the EREG/EGFR pathway by binding to AQP2 [70]), influence metabolic reprogramming (e.g., PAMP suppresses lung adenocarcinoma by regulating intracellular proline levels [71]), interfere with epigenetic modifications (e.g., AC115619-22aa impedes m^6^A methyltransferase complex assembly [67]), or regulate phenotypic switching (e.g., MFRLP reduces reactive oxygen species by binding to mitochondrial cytochrome b, thereby inhibiting vascular smooth muscle cell phenotype switching [72]). These mechanisms provide valuable references for studying micropeptide functions in oral diseases. Given that micropeptides have demonstrated clear prognostic value and targeted therapeutic potential across multiple cancers—as exemplified by miPEP205, which significantly suppresses tumor growth and metastasis and prolongs survival in tumor-bearing mice [73]—their translational prospects as novel biomarkers or therapeutic targets in oral diseases warrant in-depth exploration [61].

In summary, while specific empirical evidence for ncRNA-encoded micropeptides in oral diseases remains to be consolidated, technological innovations and cross-cancer clues have clearly illuminated their scientific and clinical potential. Future efforts should integrate oral-specific samples with multi-omics analyses and functional validation to systematically identify coding-competent ncRNAs in periodontitis, OSCC, and other oral pathologies and elucidate the biological functions of their micropeptide products, thereby opening new avenues for precision diagnosis and therapy in oral diseases.

## 6. Conclusions and Perspectives

**Figure 2:**
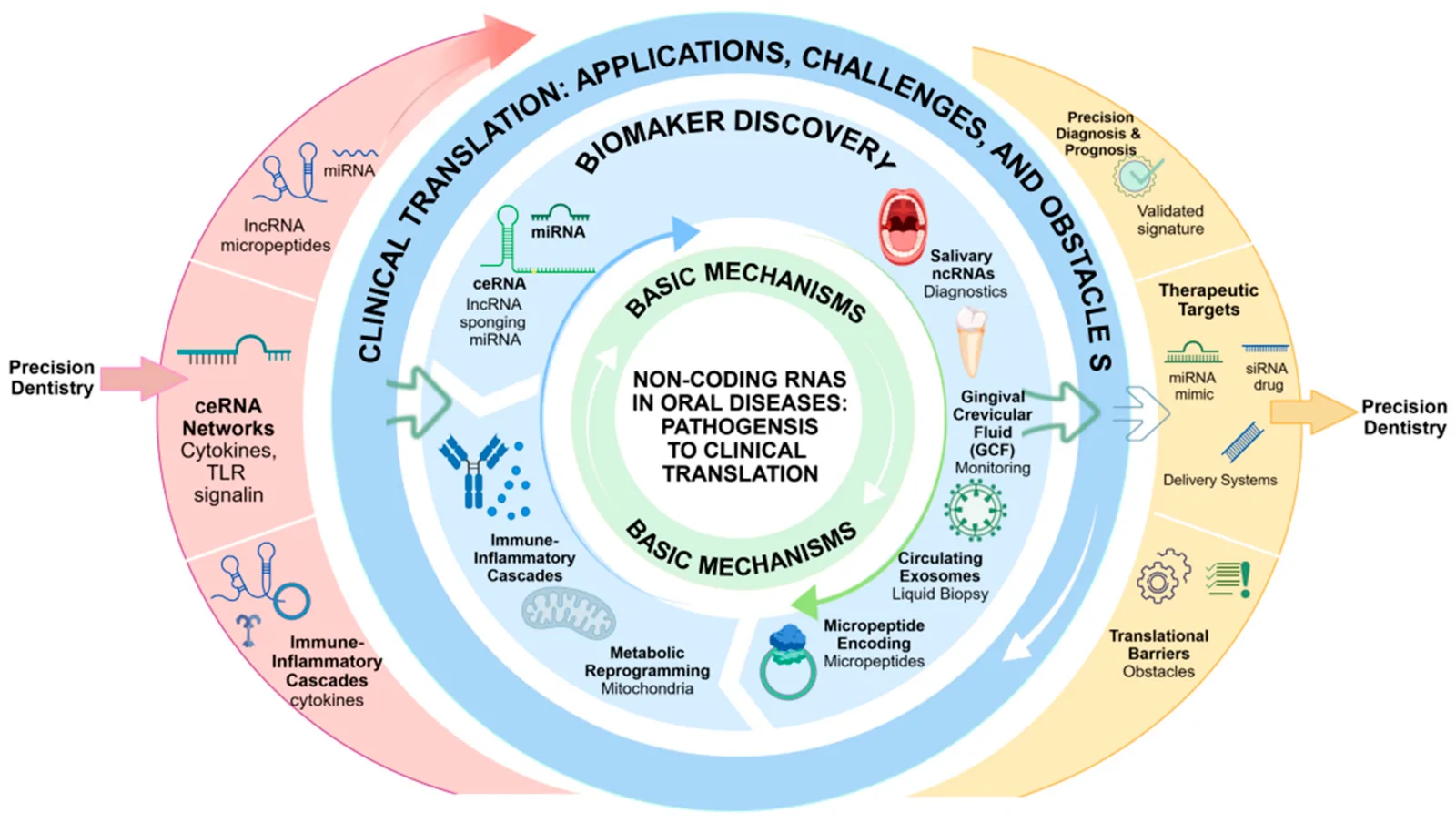
Integrative regulatory network and clinical translation framework of non-coding RNAs in oral diseases.

Overall, ncRNAs have become an important molecular layer in oral disease research. Their value lies not only in discovering new diagnostic biomarkers but also in revealing the regulatory networks connecting microbial dysbiosis, immune inflammation, tissue destruction, impaired repair, and malignant transformation in oral diseases. Research on periodontitis emphasizes the regulation of inflammatory responses and bone homeostasis by ncRNAs; studies on pulp and periapical diseases suggest ncRNA involvement in infectious inflammation and hard tissue repair; research on peri-implantitis indicates that ncRNA networks can reflect the molecular heterogeneity of material interface-associated inflammation; and studies on oral cancer demonstrate the close association of ncRNAs with tumor proliferation, metastasis, prognosis, and treatment resistance (Figure 2).

Future research should transition from descriptive studies on a single disease, single molecule, or single time point toward integrated studies across diseases, cell types, and omics layers. Single-cell transcriptomics and spatial transcriptomics can be used to dissect the specific expression of ncRNAs in epithelial cells, fibroblasts, immune cells, endothelial cells, and stem/progenitor cells; exosomal omics and metatranscriptomics can reveal RNA-level interactions between host cells and oral microbiota; and long-read sequencing and Ribo-seq can further identify lncRNA/circRNA isoforms and their potential encoded peptides. These technologies will help construct regulatory maps closer to the true state of the disease.

Regarding clinical translation, efforts should focus on standardizing ncRNA detection in saliva, GCF, and exosomes; establishing multicenter prospective cohorts; and integrating ncRNAs with the microbiome, inflammatory cytokines, imaging, pathological subtypes, and clinical risk factors. In terms of therapy, exploration of local delivery of RNA drugs, engineered exosomes, and biomaterial composite systems in periodontal regeneration, pulp repair, and adjunctive therapy for oral cancer is warranted. Only when ncRNA research simultaneously meets the four conditions of reliable mechanisms, stable detection, clinical reproducibility, and actionable intervention can it truly enter the system of precision diagnosis and treatment for oral diseases.

## Figures and Tables

**Table 1:** Summary of key ncRNAs and their regulatory mechanisms in periodontitis.

ncRNA Type	ncRNA Name	Target/Interacting Molecules	Functional Role	Signaling Pathway/Mechanism
miRNA	miR-671-5p	THBS1	Involved in diagnosis and potential therapeutic targeting of periodontitis	Regulation of THBS1 expression
	miR-146a, miR-142-3p	Inflammatory mediators	Consistently upregulated in periodontitis; modulate immune–inflammatory responses	TLR/NF-κB pathway modulation
	miR-214-3p	YAP1 (sponged by EPB41L4A-AS1)	Regulates periodontal ligament cell injury and osteogenic differentiation	EPB41L4A-AS1/miR-214-3p/YAP1 axis
	miR-181a-5p	SPP1 (sponged by AC114812)	Modulates inflammatory response of periodontal ligament cells	AC114812/miR-181a-5p/SPP1 axis
	miR-527	Caspase-5 (sponged by circ-0138959)	Participates in pyroptosis of gingival fibroblasts	circ_0138959/miR-527/caspase-5 axis
lncRNA	EPB41L4A-AS1	miR-214-3p/YAP1	Regulates periodontal ligament injury and osteogenic differentiation	ceRNA network (sponging miR-214-3p)
	AC114812	miR-181a-5p/SPP1	Modulates inflammatory response in periodontal ligament cells	ceRNA network (sponging miR-181a-5p)
	Platr3	NUDT21/NF-κB	Mediates *P. gingivalis*-suppressed cementoblast mineralization	Platr3/NUDT21/NF-κB axis
circRNA	circ_0138959	miR-527/caspase-5	Promotes pyroptosis of gingival fibroblasts	Sponges miR-527, upregulates caspase-5
	Multiple dysregulated circRNAs	Associated with Redondoviridae infection	Altered circRNA expression profile in periodontitis; potential biomarkers	Modulate ceRNA networks, involved in immune inflammation

**Table 2:** Summary of key ncRNAs and their regulatory mechanisms in oral squamous cell carcinoma (OSCC).

ncRNA Type	ncRNA Symbol	Target/Interacting Molecules	Functional Role	Signaling Pathway/Mechanism
miRNA	miR-31	Multiple oncogenes/tumor suppressors	Upregulated in saliva/plasma; correlates with TNM stage, metastasis, and chemoresistance	Promotes proliferation, invasion, and EMT
	miR-21, miR-375	Various target mRNAs	Regulate tumor proliferation, invasion, and prognosis	Classical oncogenic or tumor-suppressive miRNAs
	miR-203, miR-205	Targets involved in lymph node metastasis	High sensitivity/specificity for detecting HNSCC lymph node metastases	Regulation of metastasis-related genes
lncRNA	HOTAIR	Binds to PRC2 complex	Overexpression associated with poor overall survival, advanced TNM stage, and lymph node metastasis	Epigenetic regulation (H3K27me3)
	MALAT1	β-catenin, NF-κB	Promotes EMT, tumor growth and metastasis; independent prognostic factor	β-catenin/NF-κB pathways
	PVT1	miR-150-5p/GLUT-1, miR-7-5p/CDKL1	Promotes proliferation, invasion, and migration; inhibits apoptosis; correlates with T stage and clinical stage	Wnt/β-catenin pathway, metabolic reprogramming (glycolysis)
	H19	Multiple miRNA sponges	Involved in tumor progression; potential prognostic marker	ceRNA network, epigenetic modulation
circRNA	circRNF13	IGF2BP1 (m6A-dependent)	Promotes cisplatin resistance; positively correlates with tumor grade and stage	Enhances ITGB1 mRNA stability, driving chemoresistance
	circFNDC3B	Promotes vascular formation	Significantly upregulated; closely associated with lymph node metastasis	Promotes angiogenesis and metastasis
	circ_0001874, circ_0001971	Salivary circRNA combination	Combined diagnostic AUC = 0.922 for OSCC	Non-invasive biomarker panel
	Multiple dysregulated circRNAs	Form ceRNA networks	Construct prognostic signatures; associated with immune infiltration	Regulate VEGFA, MMPs, PI3K/AKT, and Wnt/β-catenin pathways

## Data Availability

No new data were created or analyzed in this study. Data sharing is not applicable to this article.
